# Development and evaluation of an immunochromatography-based point-of-care test kit for a rapid diagnosis of human cysticercosis

**DOI:** 10.1016/j.fawpar.2023.e00211

**Published:** 2023-10-09

**Authors:** Lakkhana Sadaow, Patcharaporn Boonroumkaew, Rutchanee Rodpai, Penchom Janwan, Oranuch Sanpool, Tongjit Thanchomnang, Yasuyuki Morishima, Marcello Otake Sato, Yasuhito Sako, Kaoru Kobayashi, Misako Iwai, Wanchai Maleewong, Hiroshi Yamasaki, Pewpan M. Intapan

**Affiliations:** a Mekong Health Science Research Institute, Khon Kaen University, Khon Kaen 40002, Thailand; b Department of Parasitology, Faculty of Medicine, Khon Kaen University, Khon Kaen 40002, Thailand; c Department of Medical Technology, School of Allied Health Sciences, Walailak University, Nakhon Si Thammarat 80161, Thailand; d Department of Parasitology, Faculty of Medicine, Mahasarakham University, Maha Sarakham 44000, Thailand; e Department of Parasitology, National Institute of Infectious Diseases, Tokyo 162-8640, Japan; f Division of Global Environment Parasitology, Faculty of Medical Technology, Niigata University of Pharmacy and Medical and Life Sciences, Niigata 956-8603, Japan; g Division of Parasitology, Department of Infectious Diseases, Asahikawa Medical University, Asahikawa 078-8510, Japan; h Division of Planning and Development, Adtec Inc., Oita 879-0453, Japan

**Keywords:** Human cysticercosis, *Taenia solium*, Immunochromatography, Antibody detection, Diagnostics, A point-of-care test

## Abstract

Human cysticercosis is a life-threatening zoonotic disease caused by infection with larvae (cysticerci) of the pork tapeworm, *Taenia solium*. This can affect the nervous system causing chronic headache and intracranial hypertension, potentially leading to epileptic seizures and paralysis. The disease is found in developing countries, especially in Southeast and South Asia, Sub-Saharan Africa, and Central and South America where porcine cysticercosis is endemic and people have a habit of eating undercooked pork. An immunochromatography-based test (ICT) kit, using *T. solium* cyst fluid as antigen, was manufactured to detect anti-*T. solium* IgG antibodies in human serum. To evaluate the kit, we used 187 serum samples including 24 from proven/confirmed cysticercosis cases, 133 from cases with other parasitosis and 30 healthy controls. Diagnostic efficiencies were calculated. The sensitivity, specificity, and accuracy were 83.3%, 92.0%, and 90.9%, respectively. Moreover, the ICT was positive before treatment but became negative after treatment, implying that this kit is also useful for follow-up monitoring post-treatment. In conclusion, we have successfully developed and present preliminary evaluation of an easy-to-handle rapid diagnostic tool for human cysticercosis in the form of an ICT platform using as antigen fluid from *T. solium* cysticerci.

## Highlights

• Development of immunochromatographic POC test tool for human cysticercosis diagnosis.
• Rapid detection of anti-*T. solium* IgG antibodies in human sera.
• Highly sensitive and specific tool for the point-of-care serodiagnosis.
• Usefulness for clinical diagnosis, follow up of post-treatment and epidemiological surveys.

## 1 Introduction

Cysticercosis is a life-threatening zoonosis caused by infection with larvae (cysticerci) of the pork tapeworm, *Taenia solium*. Human cysticercosis is found in developing countries, especially Southeast and South Asia, Sub-Saharan Africa, and Central and South America where porcine cysticercosis is endemic and people often eat poorly cooked pork (World Health Organization, 2022). In countries and regions where cysticercosis is not transmitted, imported cases are increasingly reported in international travelers (Yamasaki, 2013; Stelzle et al., 2023).

The life cycle of *T. solium* is maintained with pigs as the intermediate host and humans as the definitive host. Pigs become infected by ingesting gravid, egg-filled proglottids excreted in the stools of persons who harbor adult *T. solium* worms. Oncospheres hatching from the eggs in the intestine migrate via blood and lymph stream and develop into cysticerci in the tongue, brain, and muscles. As in pigs, humans can also be infected with *T. solium* by ingesting its eggs, thus acting as intermediate hosts, and the cysticerci develop in the central nervous system and systemic musculature (Garcia et al., 2020). Autoinfection with eggs can also occur in persons infected with adult *T. solium* (Saeed et al., 2017).

Neurocysticercosis (NCC) occurs when the central nervous system is affected by *T. solium* cysticerci and often leads to neurological disorders such as epileptic seizures and paralysis (Singh et al., 2013). In fact, it has been reported that NCC is the cause of 30% of epilepsy cases in endemic areas where people and free-roaming pigs live in close proximity (World Health Organization, 2022). Other forms of cysticercosis are subcutaneous cysticercosis (SCC), characterized by immobile nodules in the systemic musculature, including extremities, and ocular or orbital cysticercosis when eyes are infested with cysticerci (Pujari et al., 2022).

Early diagnosis of human cysticercosis is important for appropriate treatment of the disease and for prevention of severe clinical manifestations (Sato et al., 2015). Diagnosis is usually based on neuroimaging by computed tomography (CT), magnetic resonance imaging (MRI), serology, and pathology (Carpio et al., 2016). Several immunodiagnostic methods, such as ELISA and immunoblot assay, have been developed using crude or partially purified antigens of *T. solium* cyst fluid or cyst tissue extract (Garcia et al., 2020), recombinant antigens (Sako et al., 2006), and peptide antigens (Intapan et al., 2008). However, these methods are time consuming and expensive, require sophisticated equipment and infrastructure, and trained personnel not available in resource-limited settings.

Recently, a point-of-care test (POCT) was developed for the detection of *T. solium* taeniasis and NCC in hospital-based settings (Trevisan et al., 2021; Mubanga et al., 2021). Their prototype lateral-flow assay kit consisted of two strips and used two kinds of recombinant proteins, rT24H and rES33. However, the diagnostic performance of this kit was suboptimal, especially in the titration of antigen concentration (Mubanga et al., 2021). Here, we report development and diagnostic efficiencies of another immunochromatography-based POCT kit for diagnosis of human cysticercosis using *T. solium* cyst fluid as the antigen source. The new test was developed and subjected to a preliminary assessment of its sensitivity and specificity under artificial laboratory conditions and using a defined set of laboratory samples for a diagnostic case-control study.

## 2 Materials and methods

### 2.1 Parasite and preparation of antigen

*Taenia solium* cysticerci were collected from a necropsied pig in Piauí State, Brazil and were identified as *T. solium* (American genotype) by sequencing of the mitochondrial cytochrome *c* oxidase subunit I gene (Sato et al., 2006). For preparation of the antigen, cyst fluid was collected by aspiration using a 1-mL syringe from individual cysticerci isolated from the infected pig. This yielded a total of approximately 50 mL of fluid that was centrifuged and the supernatant fluid was further processed at Adtec Inc. (Oita, Japan) for use as antigen. Details of the processing done by Adtec Inc. are commercial secrets and cannot be provided here. The presence of immunogenic components with molecular masses of 10–15, 25 and 50 kDa was confirmed by SDS-polyacrylamide gel electrophoresis as previously reported (Tsang et al., 1989). The final concentration of the antigen was estimated using a Pierce BCA Protein Assay kit (Thermo Fisher Scientific, Pleasanton, CA).

### 2.2 Human sera used for evaluation

A total of 187 leftover serum samples, which had been stored at the Frozen Serum Bank, Faculty of Medicine, Khon Kaen University, Thailand and Department of Parasitology, National Institute of Infectious Diseases, Tokyo, Japan, were used for evaluation of the kit. Demographic information and diagnostic criteria relating to the *T. solium* cysticercosis patients (*n* = 24) and one additional patient with *Taenia serialis* coenurosis (Yamazawa et al., 2020), examined in the present study are presented in Table 1. Cysticercosis was diagnosed in various ways (Carpio et al., 2016; Garcia et al., 2014) including clinical signs, CT scan, MRI and ultrasonography, serological and histopathological examinations and/or molecular analysis (Table 1; Supplementary Table 1). Serum samples from cases other than cysticercosis were as follows: healthy persons who were free from any intestinal parasitic infections by stool examination and/or serologically negative against any helminth infections (10 Thai and 20 Japanese individuals); sparganosis (*n* = 22, including 1 cerebral sparganosis); cystic echinococcosis (*n* = 30) and alveolar echinococcosis cases (*n* = 6) which were diagnosed by ultrasonography and serology (Yu et al., 2008); taeniasis saginata (*n* = 5); angiostrongyliasis with eosinophilic meningoencephalitis (*n* = 16); gnathostomiasis (n = 5); toxocariasis (*n* = 10) (Yamasaki et al., 2000); trichinosis (n = 5); fascioliasis gigantica (*n* = 12); paragonimiasis (*n* = 15, including an ectopic cerebral paragonimiasis case due to *Paragonimus westermani*); amoebiasis (four cases with amoebic liver abscess and one cerebral amoebiasis case) (Janwan et al., 2022); toxoplasmosis (n = 1). The use of human serum was approved by the Ethics Committee for Human Research, Khon Kaen University (HE 621265) and the Medical Ethics Committee of the National Institute of Infectious Diseases, Tokyo, Japan (Nos. 177, 589).

**Table 1:** Demographic and clinical information about patients with cysticercosis and coenurosis who supplied sera examined in the present study.

Case No.	Year	Patient Age/ Gender/Nationality	Types	Diagnostic criteria	Suspected origin of infection	Treatments	References
Cc1	1994	72/Female/Japanese	NCC (racemose-type)	CT, MRI, Serology, pathology	China	Surgery	Yamasaki et al. (1994)
Cc2	2008	39/Female/Japanese	NCC (multiple)	CT, MRI, PET, Serology	Thailand, Lao PDR, or Madagascar	Data not available	Yamasaki (2013)
Cc3	2009	24/Male/Japanese	Ocular cysticercosis and *T. solium* taeniasis	Fundoscopy, US, Serology	Malawi	Data not available	Masuda et al. (2009)
Cc4	2009	20/F/Japanese	NCC (multiple), *T. solium* taeniasis	CT, MRI, Serology, DNA analysis, Capsule endoscopy for taeniasis	India	Surgery	Yamasaki (2013)
Cc5	2009	61/Male/Japanese	NCC (multiple, racemose type)	CT, MRI, US, Serology, Pathology, DNA analysis	India, Thailand, China or Vietnam	Surgery	Yamasaki (2013)
Cc6	2010	53/Male/Japanese	NCC (racemose-type)	CT, MRI, Serology, Pathology, DNA analysis	Japan	Surgery	Yamasaki et al. (2010)
Cc7	2010	46/Female/Chinese	NCC (multiple), SCC(multiple)	CT, MRI, US, Serology	China	Surgery	Yamasaki (2013)
Cc8	2010	31/Male/Japanese	NCC (multiple), SCC (multiple), *T. solium* taeniasis	CT, MRI, Serology, DNA analysis	India	Surgery/albendazole for cysticercosis	Kobayashi et al. (2013)
Cc9	2010	40/Male/Indian	NCC (multiple), SCC (multiple)	CT, Serology	India	Data not available	This study
Cc10	2011	35/Male/Nepalese	SCC (solitary)	CT, US, Serology, Pathology, DNA analysis	Nepal	Surgery	Yamasaki (2013)
Cc11	2012	42/Male/Indian	NCC (solitary)	Serology	India	Data not available	This study
Cc12	2014	31/Female/Cambodian	NCC (multiple) and SCC (multiple)	CT, MRI, Serology, Pathology	Cambodia	Albendazole	Sato et al. (2016)
Cc13	2016	40/Female/Nepalese	NCC (multiple)	Serology	Nepal	Albenzazole/praziquantel	This study
Cc14	2017	57/Male/Brazilian-Japanese	NCC (spinal, solitary)	Serology, Pathology, DNA analysis	Brazil	Surgery	This study
Cc15	2018	33/Male/Indian	NCC (multiple), SCC (multiple)	CT, MRI, Serology, DNA analysis	India	Data not available	This study
Cc16	2019	29/Female/Nepalese	NCC (multiple)	CT, MRI, DNA analysis, Serology	Nepal	Surgery	This study
Cc17	1987	50/Male/Thai	Ocular cysticercosis	Surgical operation and removal of eye	Thailand	Data not available	This study
Cc18	1995	72/Male/Thai	NCC (multiple), SCC (multiple)	CT, MRI	Thailand	Data not available	This study
Cc19	2004	35/Male/Thai	NCC	CT	Thailand	Data not available	This study
Cc20	2007	45/Female/Thai	NCC (multiple), SCC (left arm)	X-ray, Pathology	Thailand	Data not available	This study
Cc21	2007	39/Male/Thai	NCC (multiple)	CT, Serology	Thailand	Data not available	This study
Cc22	2008	39/Male/Thai	NCC (multiple)	MRI, Serology	Thailand	Data not available	This study
Cc23	2012	40/Male/Thai	NCC (multiple)	CT, Serology	Thailand	Data not available	This study
Cc24	2017	48/Male/Thai	NCC (multiple)	Biopsy, Pathology	Thailand	Data not available	This study
Cn1	2018	38/Male/Japanese	neurocoenurosis⁎	Pathology, DNA analysis	Japan	Surgery/albendazole	(Yamazawa et al., 2020)

NCC = neurocysticercosis, SCC = subcutaneous cysticercosis, CT = computed tomography, MRI = magnetic resonance imaging, US = ultrasonography.

⁎ neurocoenurosis due to *Taenia serialis.*

### 2.3 Immunochromatographic test (ICT) kit

To detect anti-IgG antibody against *T. solium* cysticercus antigen, an immunochromatographic device named the “iCysticercosis kit” was developed. The membrane on which antigen was sprayed, the buffer used for dilution of serum and chromatographic development, and the assay conditions were all optimized at Adtec Inc. based on results by immunoblot (LDBIO Diagnostics, Lyon, France) and ELISA at the National Institute of Infectious Diseases, Tokyo, Japan.

**Fig. 1:**
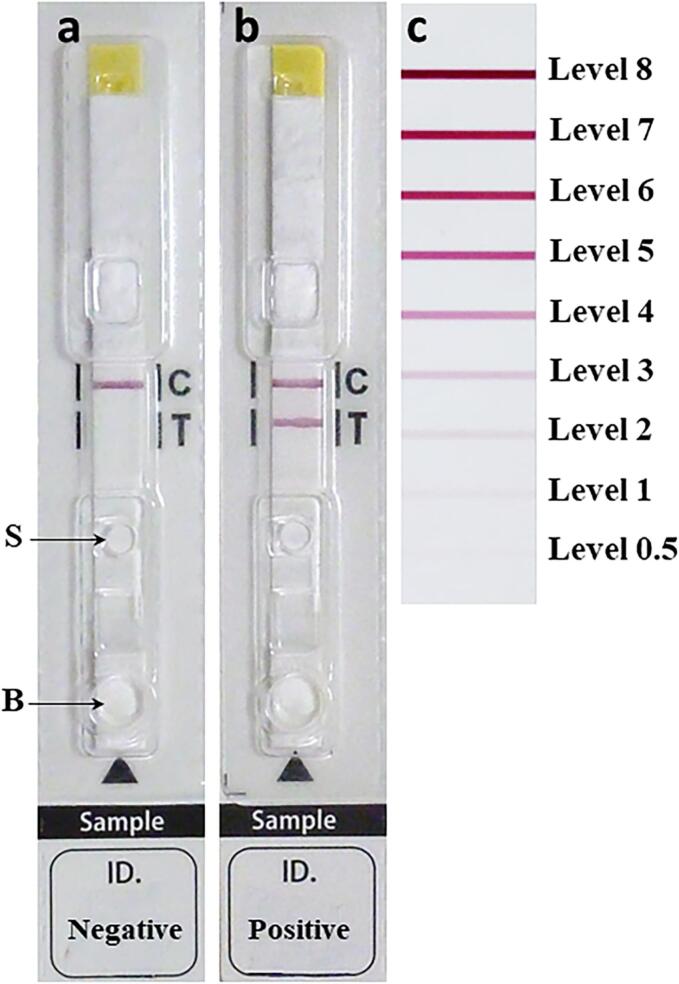
The immunochromatographic device developed in this study. Wells shown by arrows are for diluted serum sample (S) and buffer for chromatography (B). **a**, negative case with only a red band at control (C) line; **b**, positive case with red bands at both the control and test (T) lines; **c**, a card for interpretation of color intensity. (For interpretation of the references to color in this figure legend, the reader is referred to the web version of this article.)

For testing, the serum samples were diluted 1:10 with the buffer provided in the kit by Adtec Inc., and 2 μL of diluted serum sample was applied into the well inscribed with ‘S', followed by 60 μL of buffer into the well inscribed with ‘B'. Within 15 min, a red band should appear at the control C line (Fig. 1). If a red band (≥ level 0.5) also appears at the T line (Fig. 1b), the result is positive. The intensity of the red band was evaluated using a reference color board (Fig. 1c).

This study was performed according to the criteria of the STARD 2015 list for reporting diagnostic accuracy (Supplementary Table 2).

## 3 Results

**Fig. 2:**
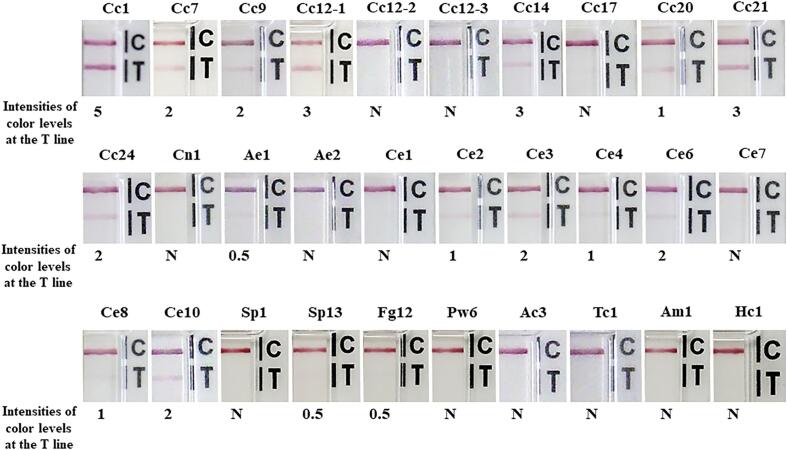
Representative results using the kit. Cc1–Cc24, cysticercosis; Cc12–1–Cc12–3, at admission, and 1 month and 5 months later after treatment; Cn1, coenurosis; Ae1 and Ae2, alveolar echinococcosis; Ce1–Ce10, cystic echinococcosis; Sp1 and Sp13, sparganosis; Fg12, fascioliasis; Pw6, paragonimiasis; Ac3, angiostrongyliasis; Tc1, toxocariasis; Am1, amoebiasis; Hc1, healthy Thai control. The color-intensity level (0.5–5) is shown below each fig. N indicates negative results.

The results are summarized in Table 2 and Supplementary Table 1. None of the 30 healthy human controls showed a positive result. Twenty out of 24 *T. solium* cysticercosis cases were positive, while one case of *T. serialis* coenorosis, which is clinically similar to cysticercosis, was negative (Table 2; Fig. 2). Interestingly, serum sample Cc12–1, collected before the patient was treated, gave a positive result, while samples Cc12–2 and Cc12–3 (from the same patient) were negative when collected at 1 and 5 months after chemotherapeutic treatment, respectively (Fig. 2). Intensity levels differed among cysticercosis cases (Table 2 and Supplementary Table 1). There were no cross-reactions between cysticercosis and other parasitic diseases requiring a differential diagnosis, such as cerebral sparganosis (no. 61, Sp6), paragonimiasis (no. 172, Pw6) and amoebiasis (no.182, Am1) (Supplementary Table 1). However, cross-reactions were observed in 13 out of 133 samples from individuals with diseases other than cysticercosis: co-infection of sparganosis and spirometrosis (*n* = 1, Sp13), alveolar echinococcosis (n = 1, Ae1), cystic echinococcosis (*n* = 10, Ce2–Ce6, Ce8–Ce12), and fascioliasis gigantica (n = 1, Fg12), (Table 2; Fig. 2; Supplementary Table 1). The sensitivity, specificity, and accuracy were 83.3% (20/24), 92.0% (150/163), and 90.9% (170/187), respectively (Table 2).

**Table 2:** Evaluation of the iCysticercosis kit using serum samples from healthy persons and patients with various parasitic infections.

**Category of serum samples**	**Number of positive cases** **/total number examined**	**Intensity of band in positive cases** **(level)**
Healthy persons	0/30	–
Cysticercosis (*Taenia solium*)	20/24	0.5–5
Coenurosis (*Taenia serialis*)	0/1	–
Sparganosis	1/22	0.5
Cystic echinococcosis	10/30	0.5–2
Alveolar echinococcosis	1/6	0.5
Taeniasis saginata	0/5	–
Angiostrongyliasis	0/16	–
Gnathostomiasis	0/5	–
Toxocariasis	0/10	–
Trichinosis	0/5	–
Fascioliasis gigantica	1/12	0.5
Paragonimiasis	0/15	–
Amoebiasis	0/5	–
Toxoplasmosis	0/1	–
**Total**	**33/187**	
Sensitivity (%) a	83.3 (62.6–95.3)	
Specificity (%) a	92.0 (86.7–95.7)	
Accuracy (%) ^a^	90.9 (85.8–94.6)	

a Numbers in parentheses indicate ranges with 95% confidence intervals.

## 4 Discussion

Currently, diagnoses of infectious diseases using chromatographic lateral-flow approaches are widely used as POCT tools and found to be suitable in a broad range of applications and are also applicable for foodborne neglected zoonotic helminthiases, including cysticercosis (Mubanga et al., 2019). Here, we report development of a promising POCT kit using *T. solium* cyst-fluid antigen from Brazil.

The positive-band intensity in our POCT did not always correlate with the type of cysticercosis (NCC, SCC and ocular type with solitary or multiple cysticerci, or cellulose- and racemose-types). One ocular cysticercosis case (Cc17) was negative, consistent with low immune response reported previously in ocular cysticercosis (Swastika et al., 2012).

Difference in antigenicity has been reported between *T. solium* from Asia (Asian genotype) and Africa/Latin America (Afro-American genotype) (Sato et al., 2006). When affinity-purified antigen from cyst fluid of *T. solium* from Asia was used, Asian cysticercosis patients showed relatively higher reactions than did cysticercosis patients in Latin America (Sako et al., 2013). In fact, the case Cc14 was a Japanese-Brazilian patient with NCC caused by a single cysticercus and yielded a relatively intense band (level 3). However, among Asian cysticercosis patients, some cases (Cc1, Cc12, Cc15, Cc21, and Cc22) were higher than other cases. The strength of the immune responses varies from person to person, and genetic factors such as immune-response genes (Mangano and Modiano, 2014) and human leukocyte-antigen (HLA) haplotypes are likely involved in the immune reactivity of cysticercosis patients.

It is unclear how long circulating antibodies last in cysticercosis patients after treatment. In one reported NCC case, the antibody responses were dramatically attenuated 20 days after chemotherapy using albendazole but tests remained positive over 4 months thereafter (Kobayashi et al., 2013). In contrast, the patient providing sample Cc12 was also treated with albendazole (Sato et al., 2016) and yielded a negative test result 1 month after treatment and remained negative 5 months after treatment. Intriguingly, a case of neurocoenurosis (Cn1) caused by *T. serialis*, which is closely related to *T. solium*, was serologically negative. However, only one case has been tested so far: a larger set of samples is required.

False-positive reactions were observed in some of cystic echinococcosis sera (Ce2–Ce12, except Ce7) and alveolar echinococcosis (Ae1). This could reflect the close phylogenetic relationship between *T. solium* and *Echinococcus* spp. One of the antigenic components of *T. solium* is a low molecular-weight hydrophilic protein which might present false-positive reactions with echinococcosis sera (Sako et al., 2000). Another possible reason for the false positives is that serum samples of cystic echinococcosis patients were collected in Qinghai Province, China where *T. solium* cysticercosis is endemic (Yu et al., 2008), and cystic echinococcosis patients might have been exposed to *T. solium* infection.

More importantly, no false-positive reactions were observed with parasitic diseases requiring diagnostic differentiation from cysticercosis, e.g. cerebral sparganosis, paragonimiasis westermani, and amoebiasis, suggesting that a differential diagnosis is possible due to differences in clinical courses and imaging findings.

In conclusion, we have successfully developed a preliminary immunochromatography based-POCT platform to diagnose human cysticercosis. The kit is promising as an easy-to-handle tool not only for supporting clinical diagnosis at the bedside and follow-up post-treatment, but also for use in large-scale sero-epidemiological surveys in remote endemic areas where medical facilities or ancillary supplies are poor. However, clinicians and laboratory technologists should be careful in diagnosing cysticercosis using this kit, particularly in areas where *T. solium* cysticercosis/taeniasis and echinococcosis are co-endemic. The test was evaluated in a laboratory setting using a defined set of samples. The performance of the test still needs to be determined in a real-world population. Sensitivity and specificity data provided here should not be accepted as generally applicable, as these characteristics depend on the population in which the test is used.

## Declaration of Competing Interest

The authors have no competing interests to declare.

## Appendix A Supplementary data

Supplementary material 1 Image 1

Supplementary material 2 Image 2
