# Supplementary material for: Development and evaluation of an immunochromatography-based point-of-care test kit for a rapid diagnosis of human cysticercosis
Source: Food Waterborne Parasitol. 2023 Oct 9;33:e00211. doi: 10.1016/j.fawpar.2023.e00211 (PMC10589370; doi:10.1016/j.fawpar.2023.e00211)
Supplement: Supplementary file 1 — Supplementary material 1 [file mmc1.docx]

**Supplementary Table 1.** Band intensity levels in each serum sample tested (n = 187), including those from healthy control individuals and individuals with various parasitic infections.

| **No.** | **Code** | **Diagnosis** | **Suspected origin**  **of infection** | **Intensity of band in positive cases (level)** |
| --- | --- | --- | --- | --- |
| 1 | Hc1 | Healthy person | Thailand | − |
| 2 | Hc2 | Healthy person | Thailand | − |
| 3 | Hc3 | Healthy person | Thailand | − |
| 4 | Hc4 | Healthy person | Thailand | − |
| 5 | Hc5 | Healthy person | Thailand | − |
| 6 | Hc6 | Healthy person | Thailand | − |
| 7 | Hc7 | Healthy person | Thailand | − |
| 8 | Hc8 | Healthy person | Thailand | − |
| 9 | Hc9 | Healthy person | Thailand | − |
| 10 | Hc10 | Healthy person | Thailand | − |
| 11 | Hc11 | Healthy person | Japan | − |
| 12 | Hc12 | Healthy person | Japan | − |
| 13 | Hc13 | Healthy person | Japan | − |
| 14 | Hc14 | Healthy person | Japan | − |
| 15 | Hc15 | Healthy person | Japan | − |
| 16 | Hc16 | Healthy person | Japan | − |
| 17 | Hc17 | Healthy person | Japan | − |
| 18 | Hc18 | Healthy person | Japan | − |
| 19 | Hc19 | Healthy person | Japan | − |
| 20 | Hc20 | Healthy person | Japan | − |
| 21 | Hc21 | Healthy person | Japan | − |
| 22 | Hc22 | Healthy person | Japan | − |
| 23 | Hc23 | Healthy person | Japan | − |
| 24 | Hc24 | Healthy person | Japan | − |
| 25 | Hc25 | Healthy person | Japan | − |
| 26 | Hc26 | Healthy persons | Japan | − |
| 27 | H27 | Healthy person | Japan | − |
| 28 | Hc28 | Healthy person | Japan | − |
| 29 | Hc29 | Healthy person | Japan | − |
| 30 | Hc30 | Healthy person | Japan | − |
| 31 | Cc1 | NCC (racemose-type) | China | 5 |
| 32 | Cc2 | NCC (multiple) | Thailand, Lao PDR,  or Madagascar | 1 |
| 33 | Cc3 | Ocular cysticercosis and *T. solium* taeniasis | Malawi | 1 |
| 34 | Cc4 | NCC (multiple), *T. solium* taeniasis | India | 1 |
| 35 | Cc5 | NCC (multiple, racemose type) | India, Thailand, China or Vietnam | − |
| 36 | Cc6 | NCC (racemose-type) | Japan | − |
| 37 | Cc7 | NCC (multiple), SCC (multiple) | China | 2 |
| 38 | Cc8 | NCC (multiple), SCC (multiple) and *T. solium* taeniasis | India | 1 |
| 39 | Cc9 | NCC (multiple), SCC (multiple) | India | 2 |
| 40 | Cc10 | SCC (solitary) | Nepal | 0.5 |
| 41 | Cc11 | NCC (solitary) | India | 2 |
| 42 | Cc12 | NCC (multiple), SCC (multiple) | Cambodia | 3 |
| 43 | Cc13 | NCC (multiple) | Nepal | − |
| 44 | Cc14 | NCC (spinal, solitary) | Brazil | 3 |
| 45 | Cc15 | NCC (multiple), SCC (multiple) | India | 3 |
| 46 | Cc16 | NCC (multiple) | Nepal | 1 |
| **No.** | **Code** | **Diagnosis** | **Suspected origin**  **of infection** | **Intensity of band in positive cases (level)** |
| 47 | Cc17 | Ocular cysticercosis | Thailand | − |
| 48 | Cc18 | NCC (multiple), SCC (multiple) | Thailand | 2 |
| 49 | Cc19 | NCC | Thailand | 2 |
| 50 | Cc20 | NCC (multiple), SCC (left arm) | Thailand | 1 |
| 51 | Cc21 | NCC (multiple) | Thailand | 3 |
| 52 | Cc22 | NCC (multiple) | Thailand | 3 |
| 53 | Cc23 | NCC (multiple) | Thailand | 0.5 |
| 54 | Cc24 | NCC (multiple) | Thailand | 2 |
| 55 | Cn1 | Neurocoenurosis due to *Taenia serialis* (proven) | Japan | − |
| 56 | Sp1 | Sparganosis (proven case) | Thailand | − |
| 57 | Sp2 | Sparganosis (proven case) | Thailand | − |
| 58 | Sp3 | Sparganosis (suspected case), ELISA antibody positive | Thailand | − |
| 59 | Sp4 | Sparganosis (proven case), Histopathology | Thailand | − |
| 60 | Sp5 | Sparganosis (proven case), Histopathology | Thailand | − |
| 61 | Sp6 | Sparganosis (cerebral type, proven case) | Japan | − |
| 62 | Sp7 | Sparganosis (proven case) | Japan | − |
| 63 | Sp8 | Sparganosis (proven case) | Japan | − |
| 64 | Sp9 | Sparganosis (proven case) | Japan | − |
| 65 | Sp10 | Sparganosis (proven case) | Japan | − |
| 66 | Sp11 | Sparganosis (proven case) | Japan | − |
| 67 | Sp12 | Sparganosis (proven case) | Japan | − |
| 68 | Sp13 | Sparganosis and spirometrosis (proven case) | Japan | 0.5 |
| 69 | Sp14 | Sparganosis (proven case) | Japan | − |
| 70 | Sp15 | Sparganosis (proven case) | Japan | − |
| 71 | Sp16 | Sparganosis (proven case) | Japan | − |
| 72 | Sp17 | Sparganosis (proven case) | Japan | − |
| 73 | Sp18 | Sparganosis (proven case) | Japan | − |
| 74 | Sp19 | Sparganosis (proven case) | Japan | − |
| 75 | Sp20 | Sparganosis (proven case) | Japan | − |
| 76 | Sp21 | Sparganosis (proven case) | Japan | − |
| 77 | Sp22 | Sparganosis (proven case) | Japan | − |
| 78 | Ce1 | Cystic echinococcosis | unknown | − |
| 79 | Ce2 | Cystic echinococcosis | Peru | 1 |
| 80 | Ce3 | Cystic echinococcosis | Afghanistan | 2 |
| 81 | Ce4 | Cystic echinococcosis | Nepal | 1 |
| 82 | Ce5 | Cystic echinococcosis | Qinghai, China | 0.5 |
| 83 | Ce6 | Cystic echinococcosis | Qinghai, China | 2 |
| 84 | Ce7 | Cystic echinococcosis | Qinghai, China | − |
| 85 | Ce8 | Cystic echinococcosis | Qinghai, China | 1 |
| 86 | Ce9 | Cystic echinococcosis | Qinghai, China | 1 |
| 87 | Ce10 | Cystic echinococcosis | Qinghai, China | 2 |
| 88 | Ce11 | Cystic echinococcosis | Qinghai, China | 1 |
| 89 | Ce12 | Cystic echinococcosis | Qinghai, China | 0.5 |
| 90 | Ce13 | Cystic echinococcosis | Qinghai, China | − |
| 91 | Ce14 | Cystic echinococcosis | Qinghai, China | − |
| 92 | Ce15 | Cystic echinococcosis | Qinghai, China | − |
| 93 | Ce16 | Cystic echinococcosis | Qinghai, China | − |
| 94 | Ce17 | Cystic echinococcosis | Qinghai, China | − |
| 95 | Ce18 | Cystic echinococcosis | Qinghai, China | − |
| **No.** | **Code** | **Diagnosis** | **Suspected origin**  **of infection** | **Intensity of band in positive cases (level)** |
| 96 | Ce19 | Cystic echinococcosis | Qinghai, China | − |
| 97 | Ce20 | Cystic echinococcosis | Qinghai, China | − |
| 98 | Ce21 | Cystic echinococcosis | Qinghai, China | − |
| 99 | Ce22 | Cystic echinococcosis | Qinghai, China | − |
| 100 | Ce23 | Cystic echinococcosis | Qinghai, China | − |
| 101 | Ce24 | Cystic echinococcosis | Qinghai, China | − |
| 102 | Ce25 | Cystic echinococcosis | Qinghai, China | − |
| 103 | Ce26 | Cystic echinococcosis | Qinghai, China | − |
| 104 | Ce27 | Cystic echinococcosis | Qinghai, China | − |
| 105 | Ce28 | Cystic echinococcosis | Qinghai, China | − |
| 106 | Ce29 | Cystic echinococcosis | Qinghai, China | − |
| 107 | Ce30 | Cystic echinococcosis | Qinghai, China | − |
| 108 | Ae1 | Alveolar echinococcosis | Japan | 0.5 |
| 109 | Ae2 | Alveolar echinococcosis | Japan | − |
| 110 | Ae3 | Alveolar echinococcosis | Qinghai, China | − |
| 111 | Ae4 | Alveolar echinococcosis | Qinghai, China | − |
| 112 | Ae5 | Alveolar echinococcosis | Qinghai, China | − |
| 113 | Ae6 | Alveolar echinococcosis | Qinghai, China | − |
| 114 | Tn1 | Taeniasis saginata | Thailand | − |
| 115 | Tn2 | Taeniasis saginata | Thailand | − |
| 116 | Tn3 | Taeniasis saginata | Thailand | − |
| 117 | Tn4 | Taeniasis saginata | Thailand | − |
| 118 | Tn5 | Taeniasis saginata | Thailand | − |
| 119 | Ac1 | Angiostrongyliasis (ocular) | Thailand | − |
| 120 | Ac2 | Angiostrongyliasis (ocular) | Thailand | − |
| 121 | Ac3 | Angiostrongyliasis (ocular) | Thailand | − |
| 122 | Ac4 | Angiostrongyliasis (ocular) | Thailand | − |
| 123 | Ac5 | Angiostrongyliasis (ocular) | Thailand | − |
| 124 | Ac6 | Angiostrongyliasis (ocular) | Thailand | − |
| 125 | Ac7 | Angiostrongyliasis (ocular) | Thailand | − |
| 126 | Ac8 | Angiostrongyliasis (ocular) | Thailand | − |
| 127 | Ac9 | Angiostrongyliasis (eosinophilic meningitis) | Thailand | − |
| 128 | Ac10 | Angiostrongyliasis (eosinophilic meningitis) | Thailand | − |
| 129 | Ac11 | Angiostrongyliasis (eosinophilic meningitis) | Thailand | − |
| 130 | Ac12 | Angiostrongyliasis (eosinophilic meningitis) | Thailand | − |
| 131 | Ac13 | Angiostrongyliasis (eosinophilic meningitis) | Thailand | − |
| 132 | Ac14 | Angiostrongyliasis (eosinophilic meningitis) | Japan | − |
| 133 | Ac15 | Angiostrongyliasis (ocular) | Taiwan | − |
| 134 | Ac16 | Angiostrongyliasis (ocular) | Japan | − |
| 135 | Gn1 | Gnathostomiasis (ocular) | Thailand | − |
| 136 | Gn2 | Gnathostomiasis (ocular) | Thailand | − |
| 137 | Gn3 | Gnathostomiasis (subcutaneous) | Thailand | − |
| 138 | Gn4 | Gnathostomiasis (ocular) | Thailand | − |
| 139 | Gn5 | Gnathostomiasis (abdominal) | Thailand | − |
| 140 | Tc1 | Toxocariasis (ocular) | Japan | − |
| 141 | Tc2 | Toxocariasis (ocular) | Japan | − |
| **No.** | **Code** | **Diagnosis** | **Suspected origin**  **of infection** | **Intensity of band in positive cases (level)** |
| 142 | Tc3 | Toxocariasis (ocular) | Japan | − |
| 143 | Tc4 | Toxocariasis (ocular) | Japan | − |
| 144 | Tc5 | Toxocariasis (visceral) | Japan | − |
| 145 | Tc6 | Toxocariasis (ocular) | Japan | − |
| 146 | Tc7 | Toxocariasis (visceral) | Japan | − |
| 147 | Tc8 | Toxocariasis (ocular) | Japan | − |
| 148 | Tc9 | Toxocariasis(ocular) | Japan | − |
| 149 | Tc10 | Toxocariasis (ocular) | Japan | − |
| 150 | Ts1 | Trichinosis (proven case) | Thailand | − |
| 151 | Ts2 | Trichinosis (proven case) | Thailand | − |
| 152 | Ts3 | Trichinosis (suspected case) | Thailand | − |
| 153 | Ts4 | Trichinosis (suspected case) | Thailand | − |
| 154 | Ts5 | Trichinosis (suspected case) | Thailand | − |
| 155 | Fg1 | Fascioliasis gigantica (proven case) | Thailand | − |
| 156 | Fg2 | Fascioliasis gigantica (proven case) | Thailand | − |
| 157 | Fg3 | Fascioliasis gigantica (proven case) | Thailand | − |
| 158 | Fg4 | Fascioliasis gigantica (proven case) | Thailand | − |
| 159 | Fg5 | Fascioliasis gigantica (proven case) | Thailand | − |
| 160 | Fg6 | Fascioliasis gigantica (proven case) | Thailand | − |
| 161 | Fg7 | Fascioliasis gigantica (proven case) | Thailand | − |
| 162 | Fg8 | Fascioliasis gigantica (proven case) | Thailand | − |
| 163 | Fg9 | Fascioliasis gigantica (proven case) | Thailand | − |
| 164 | Fg10 | Fascioliasis gigantica (proven case) | Thailand | − |
| 165 | Fg11 | Fascioliasis gigantica (proven case) | Thailand | − |
| 166 | Fg12 | Fascioliasis gigantica (proven case) | Thailand | 0.5 |
| 167 | Ph1 | Paragonimiasis (*P. heterotremus*) | Thailand | − |
| 168 | Ph2 | Paragonimiasis (*P. heterotremus*) | Thailand | − |
| 169 | Ph3 | Paragonimiasis (*P. heterotremus*) | Thailand | − |
| 170 | Ph4 | Paragonimiasis (*P. heterotremus*) | Thailand | − |
| 171 | Ph5 | Paragonimiasis (*P. heterotremus*) | Thailand | − |
| 172 | Pw6 | Paragonimiasis (cerebral, due to *P. westermani* | Japan | − |
| 173 | Pa7 | Paragonimiasis (*P. westermani* or *P. miyazakii*) | Japan | − |
| 174 | Pa8 | Paragonimiasis (*P. westermani* or *P. miyazakii*) | Japan | − |
| 175 | Pa9 | Paragonimiasis (*P. westermani* or *P. miyazakii*) | Japan | − |
| 176 | Pa10 | Paragonimiasis (*P. westermani* or *P. miyazakii*) | Japan | − |
| 177 | Pa11 | Paragonimiasis (*P. westermani* or *P. miyazakii*) | Japan | − |
| 178 | Pa12 | Paragonimiasis (*P. westermani* or *P. miyazakii*) | Japan | − |
| **No.** | **Code** | **Diagnosis** | **Suspected origin**  **of infection** | **Intensity of band in positive cases (level)** |
| 179 | Pa13 | Paragonimiasis (*P. westermani* or *P. miyazakii*) | Japan | − |
| 180 | Pa14 | Paragonimiasis (*P. westermani* or *P. miyazakii*) | Japan | − |
| 180 | Pa14 | Paragonimiasis (*P. westermani* or *P. miyazakii*) | Japan | − |
| 181 | Pa15 | Paragonimiasis (*P. westermani* or *P. miyazakii*) | Japan | − |
| 182 | Am1 | Amoebiasis (cerebral abscess) | Japan | − |
| 183 | Am2 | Amoebiasis (liver abscess) | Japan | − |
| 184 | Am3 | Amoebiasis (liver abscess) | Japan | − |
| 185 | Am4 | Amoebiasis (liver abscess) | Japan | − |
| 186 | Am5 | Amoebiasis (liver abscess) | Japan | − |
| 187 | Tp1 | Toxoplasmosis (ocular) | Japan | − |

NCC = neurocysticercosis, SCC = subcutaneous cysticercosis.
